# The risk of second malignancies following prostate cancer radiotherapy in the era of conformal radiotherapy: a statement of the Prostate Cancer Working Group of the German Society of Radiation Oncology (DEGRO)

**DOI:** 10.1007/s00066-024-02288-6

**Published:** 2024-08-28

**Authors:** C. Zamboglou, D. M. Aebersold, C. Albrecht, D. Boehmer, U. Ganswindt, N.-S. Schmidt-Hegemann, S. Hoecht, T. Hölscher, S. A. Koerber, A.-C. Mueller, P. Niehoff, J. C. Peeken, M. Pinkawa, B. Polat, S. K. B. Spohn, F. Wolf, D. Zips, T. Wiegel

**Affiliations:** 1 German Oncology Center, European University of Cyprus, 1 Nikis Avenue, 4108 Agios Athanasios, Cyprus; 2 Department of Radiation Oncology, University Hospital Freiburg, Robert-Koch-Straße 3, 79106 Freiburg, Germany; 3 Department of Radiation Oncology, Inselspital, Bern University Hospital, University of Bern, Freiburgstraße 4, 3010 Bern, Switzerland; 4 Nordstrahl Radiation Oncology Unit, Nürnberg North Hospital, Prof.-Ernst-Nathan-Str. 1, 90149 Nuremberg, Germany; 5 Charité—Universitätsmedizin Berlin, corporate member of Freie Universität Berlin and Humboldt-Universität zu Berlin, Klinik für Strahlentherapie, Hindenburgdamm 30, 12203 Berlin, Germany; 6 Department of Radiation Oncology, University Hospital Innsbruck, Anichstraße 35, 6020 Innsbruck, Austria; 7 Department of Radiation Oncology, LMU Munich, Marchioninistraße 15, 81377 Munich, Germany; 8 Department of Radiation Oncology, Ernst von Bergmann Hospital Potsdam, Charlottenstraße 72, 14467 Potsdam, Germany; 9 Department of Radiotherapy and Radiation Oncology, Faculty of Medicine and University Hospital Carl Gustav Carus, Technische Universität Dresden, Fetscherstr. 74, Dresden, Germany; 10 Department of Radiation Oncology, Heidelberg University Hospital, Im Neuenheimer Feld 400, 69120 Heidelberg, Germany; 11 Department of Radiation Oncology, Barmherzige Brüder Hospital Regensburg, Prüfeninger Straße 86, 93049 Regensburg, Germany; 12 Department of Radiation Oncology, RKH Hospital Ludwigsburg, Posilipostraße 4, 71640 Ludwigsburg, Germany; 13 Department of Radiation Oncology, Sana Hospital Offenbach, Starkenburgring 66, 63069 Offenbach, Germany; 14 Department of Radiation Oncology, TUM School of Medicine and Health, Technical University of Munich, Munich, Germany; 15 Department of Radiation Oncology, Robert Janker Hospital, Villenstraße 8, 53129 Bonn, Germany; 16 Department of Radiation Oncology, University Hospital Würzburg, Josef-Schneider-Straße 11, 97080 Würzburg, Germany; 17 Department of Radiation Oncology, Paracelsus University Hospital Salzburg, Müllner Hauptstraße 48, 5020 Salzburg, Austria; 18 Department of Radiation Oncology, University Hospital Ulm, Albert-Einstein-Allee 23, 89081 Ulm, Germany

**Keywords:** Prostate cancer, Radiotherapy, Second primary cancer

## Abstract

A significant number of prostate cancer patients are long-term survivors after primary definitive therapy, and the occurrence of late side effects, such as second primary cancers, has gained interest. The aim of this editorial is to discuss the most current evidence on second primary cancers based on six retrospective studies published in 2021–2024 using large data repositories not accounting for all possible confounding factors, such as smoking or pre-existing comorbidities. Overall, prostate cancer patients treated with curative radiotherapy have an increased risk (0.7–1%) of the development of second primary cancers compared to patients treated with surgery up to 25 years after treatment. However, current evidence suggests that the implementation of intensity modulated radiation therapy is not increasing the risk of second primary cancers compared to conformal 3D-planned radiotherapy. Furthermore, increasing evidence indicates that highly conformal radiotherapy techniques may not increase the probability of second primary cancers compared to radical prostatectomy. Consequently, future studies should consider the radiotherapy technique and other confounding factors to provide a more accurate estimation of the occurrence of second primary cancers.

## Introduction

Prostate cancer (PCa) is the most common malignancy in males in the Western world, and radiation therapy (RT) is a standard treatment option for localized or locally advanced disease. The PROTECT trial reported long-term survival rates of 15 years after RT for patients with mainly low-risk PCa [1]. The FLAME study reported 5‑year progression-free survival of 85–92% for intermediate- and high-risk patients after RT [2]. Similar biochemical failure-free survival rates of 81–95% were reported by the POP-RT trial, which included only patients with high-risk disease [3]. These results suggest that a significant number of PCa patients are long-term survivors after primary definitive therapy.

Consequently, the long-term side effects after RT for local or regional disease in PCa have gained interest in recent years. The development of a radiation-induced second primary cancer (SPC) is one of the most serious long-term consequences for these patients. A SPC is generally defined as radiation induced if it is diagnosed after a latency period (usually 3–5 years or more) following RT, occurs within or adjacent to the radiation field, is a different histological type from the original cancer, and the second tumour was not evident at the time of RT [4, 5]. Importantly, the SPC is the most common cause of death in patients with SPC [6]. In 2014, the PROBATE group of the GEC-ESTRO performed a systematic review of the risk of second malignancies after RT for PCa [7]. The authors identified 19 registry publications, 21 institutional series, and 7 other studies and concluded that the risk of SPC appears small, in the range of 1 to 220–290 over all durations of follow-up. However, they emphasized that the clinical data they found was insufficient to draw firm conclusions about the impact of more modern techniques, such as intensity modulated RT (IMRT) or brachytherapy (BT), on the incidence of SPC. In addition, most of the included studies did not account for confounders such as existing comorbidities, which hampered proper interpretation of the results.

Consequently, the Prostate Cancer Working Group of the German Society of Radiation Oncology (DEGRO) decided to collect and discuss the most current evidence on SPC by including studies that were published after the systematic review in 2014.

## Methodology

An NCBI PubMed search was performed using the following search terms: “second malignancy” AND “prostate cancer” (CZ). In addition, all members of the working group were asked to propose relevant literature. Only articles that were not included in the previous systematic review of the PROBATE group [7], not updated by more recent articles, reported on SPC after treatment with any form of RT, and included > 50,000 PCa patients were considered relevant.

## Results

A total of six publications published between 2021 and 2024 were included in the qualitative review process (Table 1).

**Table 1:** Overview of studies on SPCs after radiotherapy for prostate cancer

Paper and year of publication	Number of patients	Database (timeframe)	Median follow-up	RT technique	RT fields	Total dose and fractionation	Co-founders	Main finding(s)
*E.D. Dee et al., 2021 *[8]	303,432 in total 184,187 RP 50,023 CFRT 1190 HFRT 64,897 BT 3135 SBRT	NCDB (diagnosed until 2014)	9.1 y (IQR 7.0–11.2)	CFRT and HFRT: IMRT only SBRT: unknown BT: BT with no other RT RP: RP with no other RT	PORT	CFRT: 75–86.4 Gy in 37–49 fractions HFRT: 60–70.2 Gy in 20–30 fractions SBRT: 35–50 Gy in 4–5 fractions	Information on family history, obesity, alcohol use, and smoking status not available. Information on race available	CFRT and MHRT were associated with increased probability of a SPC compared to RP. SBRT was associated with lower odds of SM compared to CF-IMRT (aOR 0.78, 95% CI 0.66–0.93, p = 0.005) but no significant difference was found when SBRT was compared to RP (aOR 0.86, 95% CI 0.73–1.03, p = 0.102) and BT (aOR 1.126, 95% CI 0.953–1.329, p = 0.163)
*H.L. Cho et al., 2022 *[9]	50,237 in total 39,338 PORT 10,899 WPRT	NCDB (diagnosed until 2014)	8.9 y (IQR 6.9–11.0)	IMRT	PORT WPRT	Prostate ± SV: 75–90 Gy Pelvic lymph nodes: 40–55 Gy	Information on family history, obesity, alcohol use, and smoking status not available. Information on race available	No association between WPRT and an increased probability of SM compared to PORT (aOR = 1.046, 95% CI 0.968–1.130)
*H.P. Backsaw et al., 2022 *[10]	143,886 in total 52,886 with RT 91,000 without RT	Veterans Affairs Corporate Data Warehouse (2000–2015)	9 y (IQR 6–13)	Not reported	Not reported	Not reported	Smoking status at diagnosis was missing in 66% in 2000. Information on family history, obesity, and alcohol use not available. Information on race and Charlson comorbidity score available	Patients with PCa who received RT were more likely to develop SPC than patients who did not receive radiotherapy, with increased risk over time (HR for 1–5, 5–10, 10–15 years: 1.24, 1.5, and 1.59, respectively)
*K.J. Pithadia et al., 2023 *[11]	111,046 in total 65,235 primary PCa survivors 45,811 non-PCa survivors with similar demographics	SEER (2002–2015)	4.6 y (range 0–12)	IMRT 3D-conformal RT	Not reported	Not reported	Information on race and Charlson comorbidity score available. Information on family status, smoking history, and obesity not available	IMRT for prostate cancer is not associated with an increased risk of second primary cancers, either solid or hematologic. The overall HR for IMRT vs. 3DCRT was 0.91 (95% CI 0.83–0.99). This inverse association was restricted to the earlier calendar year period of prostate cancer diagnosis (HR2002–2005 = 0.66 vs. HR2006–2010 = 1.06)
*S. Monda et al., 2023 *[12]	261,609 in total 135,024 RP 67,512 EBRT 33,756 BT 16,878 EBRT + BT 8439 RP + EBRT	SEER (2000–2018)	11.6 y	Not reported	Not reported	Not reported	Information on family history, obesity, alcohol use and, smoking status not available. Information on race available	All radiation groups were associated with bladder cancer diagnosis; HR = 1.72, 1.85, 1.80, and 1.53 for EBRT, BT, EBRT + BT, and RP to EBRT, respectively, using RP as a reference (all *p* < 0.001). After radiation, bladder cancers are more likely to be sarcomatoid variant and present as muscle-invasive
*E.K. Liu et al., 2024 *[14]	569,167 in total 118,812 with RT 380,355 non-RT	SEER (1975–2016)	7.4 y	Not reported	Not reported	Not reported	Information on family history, obesity, alcohol use, race, comorbidities and, smoking status not available	RT was associated with a significantly higher incidence of SPC (1.1% vs. 1.8% at 25 years). SPC occurrence is significantly associated with worse survival (adjusted HR: 1.76)

**Abbreviations: ***aOR* adjusted odds ratio, *RP* radical prostatectomy, *RT* radiotherapy, *IMRT* intensity-modulated radiotherapy, *CFRT* conventionally fractionated radiotherapy, *HFRT* hypo-fractionated radiotherapy, *BT* brachytherapy, *SPC* second primary cancer, *SBRT* stereotactic body radiotherapy, *PCa* prostate cancer, *SEER* Surveillance, Epidemiology and End Results, *NCDB* National Cancer Database, *HR* hazard ratio, *IQR* interquartile range, *y* years

The retrospective study by Dee et al. [8] included 303,432 patients diagnosed with PCa before 2014 from the National Cancer Database in the USA. The aim of this study was to assess the rate of radiation-induced SPC after different types of curative PCa treatments: radical prostatectomy (RP), conventionally fractionated RT (CFRT), hypo-fractionated RT (HFRT), stereotactic-body RT (SBRT), and brachytherapy (BT). All patients received prostate-only RT and, for the CFRT and HFRT groups, only IMRT techniques were applied. After a median follow-up of 9.1 years, crude probabilities of SPC by treatment modality were 5.2% for RP, 8.9% for CF-IMRT, 7.7% for HF-IMRT, 7.9% for BT, and 5.3% for SBRT. Post-estimation predicted probabilities of SPC by treatment modality and adjusted odds ratios (aORs) using RP as the reference were 6.0% for RP (aOR n/a), 7.1% for CF-IMRT (aOR 1.20, 95% confidence interval [CI] 1.14–1.25, *p* < 0.001), 7.3% for HF-IMRT (aOR 1.25, 95% CI 1.01–1.55, *p* = 0.045), 6.6% for BT (aOR 1.11, 95% CI 1.07–1.16, *p* < 0.001), and 5.7% for SBRT (aOR 0.95, 95% CI 0.81–1.12, *p* = 0.567). A subgroup analysis including only irradiated patients was performed using CFRT as the reference, and BT (aOR 0.89, 95% CI 0.85–0.94, *p* < 0.001) and SBRT (aOR 0.78, 95% CI 0.66–0.91, *p* = 0.002), but not HF-IMRT (aOR 1.04, 95% CI 0.83–1.29, *p* = 0.751), were associated with lower odds of developing SPC. In addition, the SPC odds were lower for SBRT vs. CF-IMRT (aOR 0.78, 95% CI 0.66–0.93, *p* = 0.005), but no significant difference was found for SBRT vs. RP (aOR 0.86, 95% CI 0.73–1.03, *p* = 0.102) on propensity score-adjusted pairwise analysis. In further pairwise subgroup analyses of the BT and SBRT cohorts, no difference in the probability of SPCs was observed between all BT vs. SBRT (aOR 1.126, 95% CI 0.953–1.329, *p* = 0.163) were observed. However, the authors described a non-significant trend, suggesting that the probabilities of SPC with LDR BT may be more pronounced than with SBRT (aOR 1.165, 95% CI 0.977–1.389, *p* = 0.090), with no difference between HDR BT and SBRT.

In a subgroup analysis of the first study, Cho et al. [9] included 50,237 patients from the US National Cancer Database with a median follow-up of 8.9 years. The aim of this study was to compare SPC rates between prostate-only RT versus whole-pelvis RT (prostate + pelvic lymphatics). All patients were treated with IMRT. Crude probabilities of SPC were 9.16% for the extended field RT and 8.88% for prostate-only RT. The authors reported no association between the inclusion of pelvic lymphatics in the RT field and an increased probability of SPC compared to prostate-only RT (aOR 1.046, 95% CI 0.968–1.130).

The study of Backsaw et al. [10] included 143,886 patients from the Veterans Affairs Corporate Data Warehouse with a median follow-up of 9 years (from 2000–2015). The objective of the study was to assess the current incidence and risk of developing a SPC after receiving RT vs. non-RT treatments for PCa. No information regarding RT technique, RT fields, or RT fractionation schedule was provided. SPCs occurred more than 1 year after PCa diagnosis in 4257 patients (3.0%), including 1955 patients (3.7%) in the RT cohort and 2302 patients (2.5%) in the cohort without RT. The median time to development of any SPC was 6 years after PCa diagnosis, and the most common SPC was bladder cancer (RT cohort: 957 patients [1.8%]; non-RT cohort: 985 patients [1.1%]) The authors also conducted a multivariate analysis and reported that patients in the RT cohort had a higher risk of SPC compared with those in the non-RT cohort 1 to 5 years after diagnosis (hazard ratio [HR] 1.24, 95% CI 1.13–1.37, *p* < 0.001), with higher adjusted HRs in the subsequent 15 years (years 5–10: 1.50, 95% CI 1.36–1.65, *p* < 0.001; years 10–15: 1.59, 95% CI 1.37–1.84, *p* < 0.001; years 15–20: 1.47, 95% CI 1.08–2.01, *p* = 0.02).

The study by Pithadia et al. [11] included 111,046 patients from the Surveillance, Epidemiology and End Results Program with a median follow-up of 4.6 years (from 2002–2015). The authors evaluated whether the RT type (IMRT vs. 3D) was associated with SPC risk among men treated for PCa. No information on treatment fields and fractionation regimen was provided. Among 5‑year survivors, 2688 men were diagnosed with a second primary solid cancer (IMRT: *n* = 1306; 3D: *n* = 1382). The overall HR for IMRT vs. 3D was 0.91 (95% CI 0.83–0.99). This inverse association was restricted to the earlier calendar year period of PCa diagnosis (2002–2005: HR 0.85, 95% CI 0.76–0.94; 2006–2010: HR 1.14, 95% CI 0.96–1.36).

Monda et al. [12] updated a study by Zhang et al. [13] and included 261,609 patients from the Surveillance, Epidemiology and End Results Program with a median follow-up of 11.6 years (from 2000–2018). The authors sought to compare the long-term risk of bladder cancer after different localized PCa treatments: RP, external-beam RT (EBRT), BT, combined EBRT + BT, and RP followed by EBRT. The authors did not provide any information regarding RT technique, RT fields, or RT fractionation schedule. Using a Cox model, the authors reported HRs of 1.72, 1.85, 1.80, and 1.53 for bladder cancer diagnosis after EBRT, BT, EBRT + BT, and RP + EBRT, respectively, using RP as the reference (all *p* < 0.001). In addition, the authors observed a 6-fold greater proportion of sarcomatoid variant in bladder cancers after RT (0.61% vs. 0.1%, *p* = 0.0076).

The study by Liu et al. [14] included 569,167 patients with a median follow-up of 7.4 years (from 1975–2016) from the Surveillance, Epidemiology and End Results Program. The authors controlled for differences in age, year of diagnosis, and surgery at time of PCa treatment and observed that the RT received was associated with a significantly higher incidence of SPCs (1.1% vs. 1.8% at 25 years). The authors emphasized that, among those who received RT during the initial PCa treatment (*n* = 195,415), developing an SPC was significantly associated with worse survival (adjusted HR 1.76), especially among younger patients (< 63 years, adjusted HR 2.36).

## Discussion

The aim of this analysis was to provide a contemporary overview of the development of SPCs in the era of modern RT in terms of IMRT/IGRT, SBRT, or BT. For this purpose, six original articles published between 2021–2024 were included.

First, we asked whether state-of-the-art RT techniques increase the risk of SPCs compared to RP. This question was addressed by four studies and, in general, after RT for PCa, the risk for SPCs is increased by approximately 0.7–1% up to 25 years after RT. Backsaw et al. [10] reported a higher SPC risk after a median follow-up of 9 years after the diagnosis of PCa for RT (3.7%) compared to RP (2.5%). This risk increased over time with a HR of 1.59 for 10–15 years after diagnosis. No information regarding RT technique was provided by the authors, and it is likely that many patients were treated with CFRT because the period for analysis was 2002–2015. Similar findings were reported by Monda et al. [12], demonstrating incidences of bladder cancer per 100 person years of 0.12, 0.22, 0.24, 0.23, and 0.15 for RP, EBRT, BT, EBRT + BT, and RP plus EBRT, respectively. Interestingly, the authors reported a higher incidence of dedicated histopathological sub-types in patients with bladder cancer following RT. Again, no information regarding RT technique was provided by the authors. Dee et al. [8] reported that HF-IMRT, CF-IMRT, and BT, but not SBRT, were associated with increased rates of SPC compared to RP, with crude probabilities of SPC of 5.2% for RP, 8.9% for CF-IMRT, 7.7% for HF-IMRT, 7.9% for BT, and 5.3% for SBRT. This differentiated approach of creating dedicated RT subgroups, including SBRT, is novel in the literature. However, considering different follow-up times (e.g., median follow-up after SBRT and BT of 7.4 and 10.1 years, respectively), the results must be interpreted with caution. Four older registry studies observed that irradiated patients were at a significantly increased risk of SPC compared to non-irradiated PCa patients [15–17]. Again, none of the studies performed a dedicated subgroup analysis looking at SBRT or HDR BT separately. The most recent study, by Liu et al. [14], reported an increased risk of SPC of 0.7% at 25 years by controlling for age differences, year of diagnosis, and tumour grade. Surely, the preferred way to answer this question is long-term follow-up data from randomized controlled trials including patients with RT and non-RT treatment options. A recent update of the PROTECT study reported the outcomes after a median of 15 years of follow-up of 545 PCa patients randomly assigned to receive active monitoring, 553 to undergo RP, and 545 to undergo EBRT [1]. Overall, approximately 10% of the patients in the PROTECT study died due to SPC, and 2.7% of the patients died due to progressive PCa [1]. The authors did not observe any significant differences regarding the occurrence rate of SPCs and SPC-associated deaths between the three study arms. Similarly, Wiltink et al. pooled > 2500 patients with endometrial cancer from the prospective PORTEC1 and 2 trials and concluded that, after a median of 13 years follow-up, patients who underwent RT had no higher probability of developing SPCs than patients who were treated with surgery alone (26.5% and 25.6% of patients had SPCs in the non-RT and RT arms, respectively) [18].

Second, we asked whether the RT technique influences the incidence of SPCs after RT. Dee et al. [8] observed that CF-IMRT possesses a significantly higher risk of SPCs compared to SBRT and BT. The authors did not clarify the applied RT technique (3D vs. IMRT) for SBRT, but most of the patients received SBRT with IMRT. These findings are comparable to older studies [19] and suggest that the more conformal dose distributions afforded by BT or SBRT play a role in lowering the probability of SPCs. Interestingly, a pairwise subgroup analysis revealed that SPC incidence is not significantly different between SBRT and BT in general. However, a non-significant trend towards a higher probability of LDR compared to HDR BT was observed. Considering comparable dose distributions between SBRT, LDR, and HDR BT, we assume that the dose rate plays a role in the development of SPCs. This is in accordance with radiobiological modelling of carcinogenesis, which suggests that SBRT may reduce the probability of SPC due to treatment in fewer fractions and a lower total dose [20, 21]. The reduction of low-dose irradiation due to less cone-beam CT imaging for image guidance in ultra-hypofractionated RT or BT was suggested by the authors as another reason for reduced SPCs. A second paper by Pithadia et al. [11] concluded that IMRT for PCa is not associated with an increased risk of SPC compared to conformal 3D-RT. The increased scatter irradiation to distant normal tissue with IMRT was considered a risk factor for the development of SPCs. However, smaller target volumes for IMRT and shorter follow-up time for patients treated with IMRT may explain the observed similarity in the occurrence of SPCs.

Finally, we asked whether the RT field influences the risk of SPCs in PCa patients. Cho et al. [9] reported no significant difference between PORT and WPRT in PCa patients treated with IMRT. The authors discussed that a greater integral dose with WPRT may theoretically increase the risk of SM, and it is possible that the relative increase from PORT to WPRT does not result in a clinically meaningful difference regarding SPC risk. Comparable results were reported by the investigators of the RTOG 9413 study using non-IMRT techniques [22].

Notably, all studies reporting on the incidence rate of SPCs were retrospective studies using large data repositories or non-planned analyses of randomized controlled trials including a limited number of patients. The rate of SPC-associated deaths was reported by the PROTECT study group [1]. In addition, there was much heterogeneity in the above studies in terms of follow-up, data sources, confounders, comparisons, and results, which makes it difficult to draw firm conclusions. For example, smoking is an important potential confounding factor, especially when considering bladder cancer. Smokers may not be eligible for surgery and, therefore, cohorts of patients treated with RT may contain a higher proportion of smokers, which will increase the risk of SPCs. Another confounder is race. Monda et al. [12] reported that Caucasian race was associated with an increased risk for bladder cancer diagnosis and death. Backshaw reported that Black race (vs. White race: HR 0.76, *p* < 0.001) was associated with a lower risk of developing SPCs [10]. However, race and other potential patient characteristics, such as the Charlson comorbidity index or visceral adipose tissue [23], were not considered in all studies (Table 1). Furthermore, there may be distinct radiobiological (e.g., genomic risk) characteristics that may lead to an increased risk of SPCs and are not yet understood. For example, Wang et al. [24] observed the highest risk of SPCs in females following RT in the head and neck area and a decreasing risk of SPC with increasing age, which is important because PCa rarely affects younger men. Considering the heterogeneous reporting of confounders, the studies’ results must be interpreted with caution.

In conclusion, PCa patients treated with curative RT have an increased overall risk of the development of SPCs of approximately 0.7–1% compared to patients treated with surgery up to 25 years after treatment. This risk increases over time with a HR of approximately 1.5, and the occurrence of SPCs is significantly associated with worse survival, especially among younger patients (< 63 years). However, current evidence suggests that the implementation of IMRT is not increasing the risk of SPCs compared to conformal 3D-RT. Even the expansion of the RT field in terms of WPRT with IMRT may not increase the risk of SPCs compared to PORT. There is also growing evidence that highly conformal RT techniques may not increase the probability of SPCs compared to RP. Similarly, we suggest considering the RT technique as a confounding factor in future studies because it is likely that the usage of SBRT will increase in the future for the treatment of primary localized or locally advanced PCa.
